# The PI3K-AKT-mTOR pathway activates recovery from general anesthesia

**DOI:** 10.18632/oncotarget.10172

**Published:** 2016-06-19

**Authors:** Yun-Hui Zhang, Jin Zhang, Jian-Nan Song, Xue Xu, Jin-Song Cai, Yang Zhou, Jin-Gui Gao

**Affiliations:** ^1^ Department of Anesthesia, the Second Hospital of Hebei Medical University, Shijiazhuang, P. R. China; ^2^ Department of Anesthesia, Shijiazhuang Obstetrics and Gynecology Hospital, Shijiazhuang, P. R. China; ^3^ Department of Anesthesia, Chifeng Municipal Hospital, Chifeng, P. R. China; ^4^ Department of Anesthesia, Hebei Medical University, Shijiazhuang, P. R. China

**Keywords:** PI3K-AKT-mTOR pathway, general anesthesia, recovery from general anesthesia, righting reflex, morris water maze, Pathology Section

## Abstract

We investigated roles of PI3K-AKT-mTOR pathway in recovery from general anesthesia. Sprague-Dawley rats divided into five groups: saline+artificial cerebrospinal fluid (ACSF; Group A), ketamine+ACSF (Group B), ketamine+IGF-1 (Group C), ketamine+PI3K inhibitor (Group D), and PI3K/Akt agonists (Group E). Proportion of δ waves on ECoGs was recorded. Rats were tested for duration of loss of righting reflex (LORR), ataxic period and behavior in Morris water maze. mRNA and protein expression of members of PI3K-AKT-mTOR pathway were measured by RT-qPCR and Western blots. Histopathologic changes in hippocampal tissues observed by HE staining. We found that the proportion of δ waves decreased in Group C, while increased in Group D compared with Group B; the durations of LORR and ataxic period were shorter in Group C, but longer in Group D. In Morris water maze, escape latency (EL) and duration and frequency of staying on platform was shorter in Group C and longer in Group D than in Group B. Group A exhibited low expression of proteins in PI3K-AKT-mTOR pathway, while p-AKT, p-mTOR and p-P70S6K expression increased in cerebral cortex, brain stem, and thalamus in Group C. By contrast, expression of those proteins was lower in Group D than Group B. Those proteins expressions were higher in Group E than in Group A. HE staining showed that anesthesia may induce cell apoptosis in rat hippocampal CA1 areas, and PI3K/Akt agonists could inhibit apoptosis. Our results suggest that activation of PI3K-AKT-mTOR pathway may promote recovery from general anesthesia and enhance spatial learning and memory.

## INTRODUCTION

In this day and age, patients are able to undergo complicated surgical procedures under novel anesthetic techniques, but new drugs may still improve the safety of these procedures [1]. General anesthesia is a drug-induced, reversible condition with specific behavioral and physiological characteristics, such as unconsciousness, akinesia, analgesia, and amnesia, accompanied by stability of the autonomic, respiratory, cardiovascular, and thermoregulatory systems [2, 3]. However, patients with general anesthesia may suffer from complications, including delayed recovery, postoperative nausea and vomiting, hypoxemia, acute atelectasis, and postoperative cognitive dysfunction [4–7]. The complications of anesthesia may prolong the length of stay and increase the cost of hospitalization, diminish the quality of the patient's life, or even, increase patient morbidity or mortality [8, 9]. In this regard, how best to promote patient's recovery from general anesthesia is a crucial research question for medicine and neuroscience.

The phosphatidylinositol-3 kinase/AKT/mammalian target of rapamycin (PI3K-AKT-mTOR) signaling pathway is crucial to various aspects of cellular maintenance, in both physiological and pathological conditions [10]. It is important for regulating the cell cycle and may be associated with cellular proliferation, migration, adhesion, metabolism, invasion, and survival [11, 12]. PI3K is a family of enzymes that phosphorylate the 3′-OH of the inositol ring of phosphatidylinositol, and includes three classes: Class I, Class II, and Class III [10]. Phosphatidylinositol 3,4,5-triphosphate (PIP3) is an important lipid second messenger generated by PI3K, which may be implicated in several signal transduction pathways, and activates the serine/threonine kinases PDKl and AKT [13, 14]. AKT, also known as protein kinase B, controls protein synthesis and cell growth by leading to the phosphorylation of mTOR [15, 16], a serine-threonine protein kinase that stimulates several intracellular processes in response to extracellular signals [17].

Activation of the PI3K-AKT-mTOR pathway may lead to a disturbance in control of cell growth and survival, and ultimately result in metastasis and angiogenesis as well as therapy resistance [10]. The PI3K-AKT-mTOR pathway is antagonized by phosphatase and tensin homolog (PTEN), glycogen synthase kinase 3β (GSK3B), and homeobox protein 9 (HB9) [18, 19]. The PI3K-AKT-mTOR pathway is also implicated in the development and progression of pathological pain, and it may be associated with the development of pain caused by tissue injury [20, 21]. However, few studies have focus on the relationship between the PI3K-AKT-mTOR pathway and recovery from general anesthesia. In order to understand the central action of the PI3K-AKT-mTOR pathway on the recovery from general anesthesia, we established an anesthetized rat model and investigated mRNA and protein expression of PI3K-AKT-mTOR pathway proteins in brain tissues.

## RESULTS

### Vital signs

All rats in Group A, B, C, D and E had smooth, tender and rosy hair and skin, and the vital signs of all rats, including HR, RR, MAP, PaO_2_, PaCO_2_ and SaO_2,_ were stable. No differences in vital signs were found among the five groups (Table 2).

**Table 1:** The primer sequences and sizes of real-time quantitative polymerase chain reaction (RT-qPCR) products

Target gene	Primer sequences	Sizes
PI3K	F: 5′-TTCTCTGATCCATTAACCTT-3′	143bp
	R: 5′-TCTTTGACAACTTGATCCTG-3′	
Akt	F: 5′-TCACCTCTGAGACCGACACC-3′	146bp
	R: 5′-ACTGGCTGAGTAGGAGAACTGG-3′	
mTOR	F: 5′-TCGGCACATCACTCCCTTCA-3′	155bp
	R: 5′-AACAACGGCTTTCCACCAGA-3′	
P70S6K	F: 5′-CTACAGAGACCTGAAGCCGGAGA-3′	114bp
	R: 5′-AATGTGTGCGTGACTGTTCCATC-3′	
β-actin	F: 5′-GGCACAGTCAAGGCTGAGAATG-3′	143bp
	R: 5′-ATGGTGGTGAAGACGCCAGTA-3′	

F: forward primer; R: reverse primer; bp: base-pairs

**Table 2:** The vital signs of rats (`x ± s)

Vital signs	Group A	Group B	Group C	Group D	Group E
HR (beats/min)	302.3 ± 17.6	305.1 ± 10.2	307.7 ± 17.4	303.3 ± 13.2	304.8 ± 15.6
RR (breaths/min)	87.0 ± 7.6	86.3 ± 7.0	87.5 ± 6.2	85.7 ± 5.9	87.0 ± 6.8
MAP (mmHg)	112.8 ± 10.6	113.4 ± 10.2	114.5 ± 13.1	111.0 ± 12.5	113.9 ± 11.8
PaO_2_ (mmHg)	79.5 ± 5.3	80.1 ± 4.8	81.2 ± 6.0	80.9 ± 1.8	82.1 ± 3.1
PaCO_2_ (mmHg)	38.8 ± 4.2	38.4 ± 4.1	39.2 ± 3.6	39.0 ± 4.1	39.7 ± 3.8
SaO_2_ (%)	98.1 ± 1.3	97.9 ± 1.1	98.2 ± 1.6	98.3 ± 1.8	99.4 ± 1.4

Note: HR, heart rate; RR, respiratory rate; MAP, mean arterial pressure; PaO_2_, arterial partial pressure of oxygen; PaCO_2_, partial artery carbon dioxide pressure; SaO_2_, arterial oxygen saturation. Group A, blank control group; Group B, anesthetized rat model group; Group C, anesthetized rat model+PI3K/Akt agonists group; Group D, anesthetized rat model+PI3K/Akt antagonists group; Group E: PI3K/Akt agonist group.

### The proportion of δ wave in ECoG

**Figure 1:**
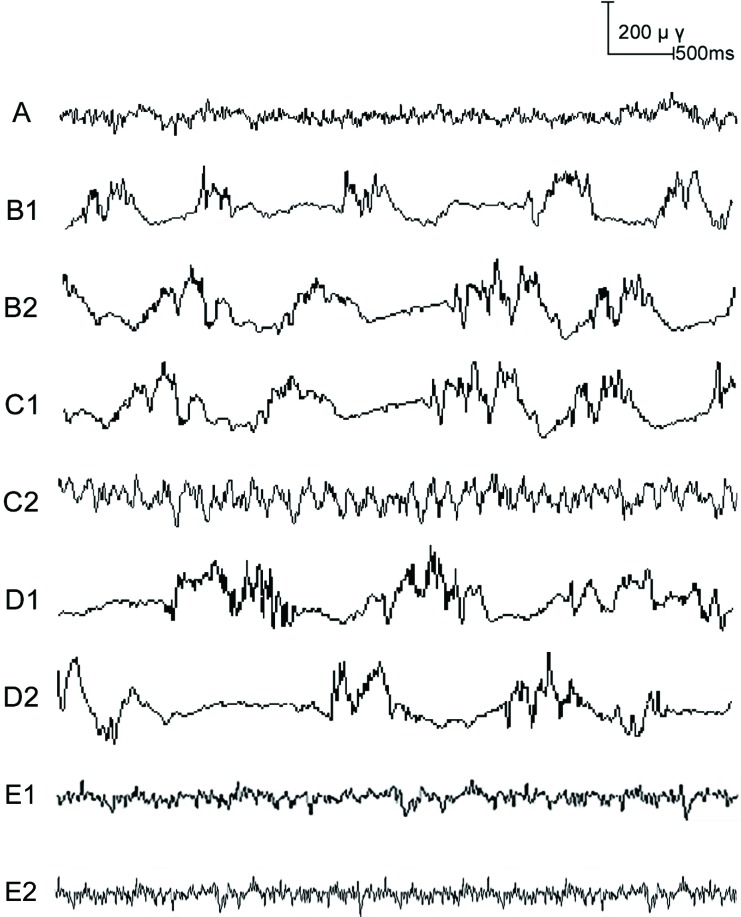
The dynamic changes of electrocorticogram (ECoG) in rats before and after drug administration Note: Group **A.**, blank control group; Group **B.**, anesthetized rat model group; Group **C.**, anesthetized rat model + PI3K/Akt agonist group; Group **D.**, anesthetized rat model + PI3K/Akt antagonists group; Group **E**. PI3K/Akt agonists group A: ECoG image of rats in Group A; B1-B2: ECoG image of rats in Group B at the time points of 5 min before (B1) and after (B2) drug administration; C1-C2: ECoG image of rats in Group C at the time points of 5 min before (C1) and after (C2) drug administration; D1-D2: ECoG image of rats in Group D at the time points of 5 min before (D1) and after (D2) drug administration; E1-E2: ECoG image of rats in Group E at the time point of 5 min before (E1) and after (E2) drug administration.

**Figure 2:**
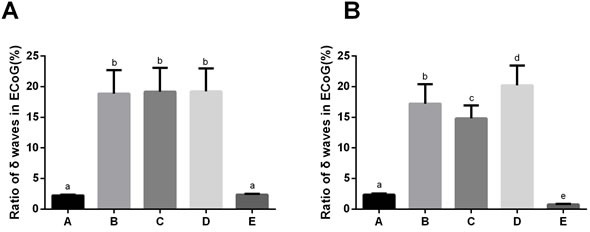
The proportion of δ waves in electrocorticogram (ECoG) of rats in the five groups before and after drug administration Note: Group A, blank control group; Group B, anesthetized rat model group; Group C, anesthetized rat model + PI3K/Akt agonist group; Group D, anesthetized rat model + PI3K/Akt antagonists group; Group E: PI3K/Akt agonist group. A: 5 min before drug administration; B: 5 min after drug administration; The superscript lowercase letters (^a, b, c, d, e^) represent the pairwise comparison among the five groups with the same protein and tissue. The same superscript lowercase letter indicated the *P* > 0.05, and the different superscript lowercase letter indicated the *P* < 0.05.

The ECoG of rats was measured 5 min before and after drug administration. Compared with Group A, the proportion of δ waves in ECoG was higher in Groups B, C and D both 5 min before and 5 min after drug administration (all *P* < 0.05). At 5 min before drug administration, the proportion of δ wave in ECoG in Groups B, C, and D did not differ from each other (Group B: 18.87 ± 3.82%; Group C: 19.18 ± 3.91%; Group D: 19.21 ± 3.75%). And the proportion of δ wave in ECoG in Group A and Group E also showed no significant differences (Group A: 2.24 ± 0.13% *vs*. Group E: 2.36 ± 0.14%; *P* > 0.05). At 5 min after drug administration, the proportion of δ wave in ECoG in Group E decreased significantly compared with Group A (Group E: 0.76 ± 0.08% *vs*. Group A: 2.24 ± 0.13%; *P* < 0.05). As compared to Group B, the proportion of δ waves in ECoG in Group C was decreased 5 min after drug administration (14.82 ± 2.13% *vs*. 17.24 ± 3.15%; *P* < 0.05). In contrast, the proportion of δ waves in ECoG in Group D was increased 5 min after drug administration when compared to Group B (20.24 ± 3.19% *vs*. 17.24 ± 3.15%; *P* < 0.05; Figures 1 and 2).

### The duration of LORR and ataxic period of anesthetized rats

**Figure 3:**
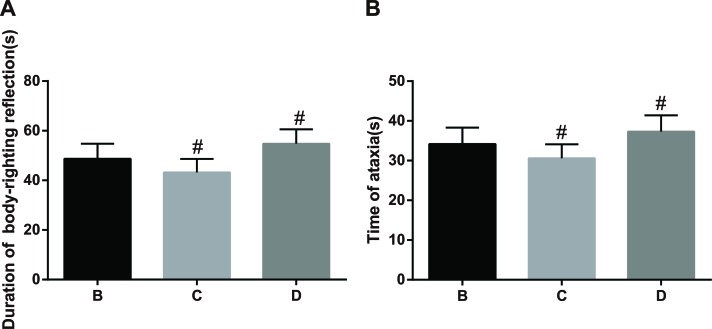
The comparisons of the duration of loss of righting reflex (LORR) and ataxic period of anesthetized rats among the three groups Note: Group B, anesthetized rat model group; Group C, anesthetized rat model + PI3K/Akt agonist group; Group D, anesthetized rat model + PI3K/Akt antagonists group; A: comparison of the duration of LORR; B: comparison of the ataxic period; ^#^, compared with Group B, *P* < 0.05.

The LORR of rats in Groups B, C and D were measured within 120 s after i.p. injection of ketamine. The duration of LORR and the ataxic period of rats in Group C were shorter than those in Group B (43.2 ± 5.4 *vs*. 48.7 ± 6.1; *P* < 0.05); while, the duration of LORR and the ataxic period of rats in Group D were longer as compared to Group B (54.8 ± 5.8 *vs*. 48.7 ± 6.1; *P* < 0.05, Figure 3).

### The effects of the PI3K-AKT-mTOR pathway on spatial learning and memory of anesthetized rats

**Figure 4:**
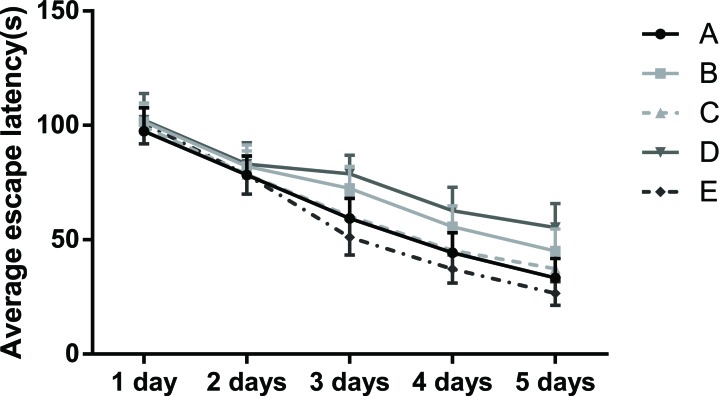
Graph of the average escape latency (EL) of rats in the place navigation test Note: Group A, blank control group; Group B, anesthetized rat model group; Group C, anesthetized rat model + PI3K/Akt agonist group; Group D, anesthetized rat model + PI3K/Akt antagonists group; Group E, PI3K/Akt agonist group.

In the PNT the EL in all five groups were decreased with training, and the EL was stable from the third day. There were no differences in EL of among the five groups on Day 1 and Day 2. However, as compared to Group A, the EL of rats in Group B and Group D were longer on Days 3-5 (all *P* < 0.05), while no differences were observed between Group A and Group C, and the EL of rats in Group E significantly shortened when compared with that in Group A (*P* < 0.05). Further, as compared to Group B, the EL of rats in Group C was shorter, while the EL of rats in Group D was longer from Days 3-5 (all *P* < 0.05; Table 3 and Figure 4).

**Table 3:** The comparison of escape latency (EL) in place navigation test in five groups (`x ± s)

	Day 1	Day 2	Day 3	Day 4	Day 5
Group A	97.48 ± 10.16^a^	78.34 ± 8.25^a^	59.28 ± 8.82^a^	44.36 ± 8.74^a^	33.34 ± 8.45^a^
Group B	101.52 ± 8.23^a^	82.06 ± 9.37^a^	72.39 ± 9.73^b^	55.74 ± 9.05^b^	45.07 ± 9.61^b^
Group C	99.47 ± 9.88^a^	78.56 ± 10.27^a^	60.36 ± 9.25^a^	45.30 ± 8.64^a^	37.27 ± 6.73^a^
Group D	102.37 ± 11.62^a^	83.04 ± 9.36^a^	78.82 ± 8.07^c^	62.71 ± 10.26^c^	55.35 ± 10.44^c^
Group E	100.87 ± 8.89^a^	78.29 ± 8.34^a^	51.04 ± 7.76^d^	37.24 ± 6.23^d^	26.52 ± 5.18^d^

Note: Group A, blank control group; Group B, anesthetized rat model group; Group C, anesthetized rat model+PI3K/Akt agonists group; Group D, anesthetized rat model+PI3K/Akt antagonists group; Group E: PI3K/Akt agonist group. The superscript lowercase letters (^a^, ^b^, ^c^, ^d^) represent the pairwise comparison among the five groups in the same day. The same superscript lowercase letter in same column indicated the *P* > 0.05, and the different superscript lowercase letter in same column indicated the *P* < 0.05.

**Figure 5:**
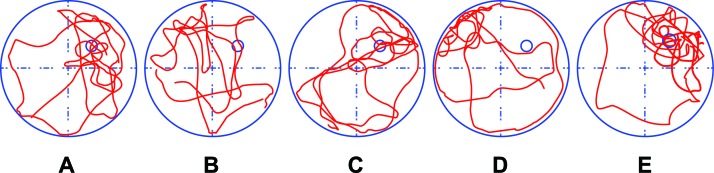
The motion curves of rats in the spatial probe test (SPT) of rats in the five groups Note: Group A, blank control group; Group B, anesthetized rat model group; Group C, anesthetized rat model + PI3K/Akt agonist group; Group D, anesthetized rat model + PI3K/Akt antagonists group; Group E, PI3K/Akt agonist group.

The SPT was performed to test the long-term memory of the rats. The frequency of passing the platform and the duration of staying in the quadrant of rats in Group B and Group D were lower, while in Group E were higher than those in Group A (all *P* < 0.05) No differences were observed between Group A and Group C. As compared to Group D, the frequency of passing the platform and the duration of staying in the quadrant of rats in Group B were higher (both *P* < 0.05; Table 4). Motion curves showed that the rats in Group A, Group C and Group E had clear and intentional movements to the original platform, suggesting that the rats had good memory. The motion curves of rats in Group B and Group D were unclear with unintentional movements to the original platform, suggesting that the rats had relatively weak memory (Figure 5).

**Table 4:** The comparisons on the frequency of passing platform and the duration of stay in the spatial probe test (SPT) among the five groups (`x ± s)

	Group A	Group B	Group C	Group D	Group E
The duration of stay in SPT (s)	32.34 ± 6.24^a^	18.27 ± 4.91^b^	31.22 ± 4.10^a^	12.36 ± 3.12^c^	36.28 ± 6.40^d^
The frequency of passing platform in SPT (n)	11.23 ± 1.63^a^	5.87 ± 0.43^b^	11.10 ± 1.54^a^	0.17 ± 0.38^c^	14.23 ± 2.06^d^

Note: Group A, blank control group; Group B, anesthetized rat model group; Group C, anesthetized rat model+PI3K/Akt agonists group; Group D, anesthetized rat model+PI3K/Akt antagonists group; Group E: PI3K/Akt agonist group. The superscript lowercase letters (^a^, ^b^, ^c^, ^d^) represent the pairwise comparison among the five groups. The same superscript lowercase letter indicated the *P* > 0.05, and the different superscript lowercase letter indicated the *P* < 0.05.

In the VPT the average EL for Groups A, B, C, D and E were 7.95 ± 1.39 s, 8.26 ± 2.02 s, 8.08 ± 2.17 s, 8.33 ± 1.78 s, and 7.89 ± 1.26 s, respectively. No differences were found among the five groups, indicating that there was no difference in the sensorimotor functions among the groups that would negatively impact spatial learning or memory.

### PI3K-AKT-mTOR pathway mRNA expression

**Figure 6:**
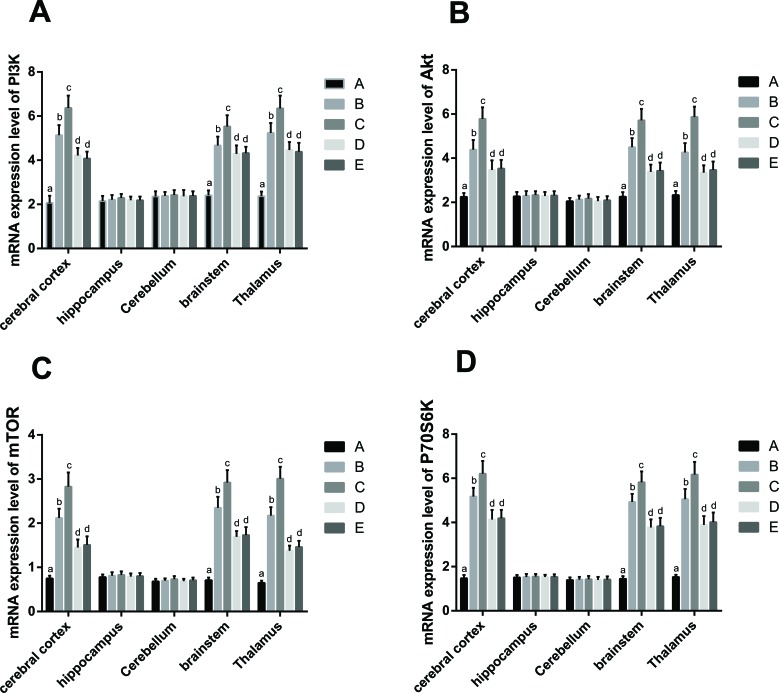
The mRNA expression in the PI3K-AKT-mTOR pathway in different tissues Note: Group A, blank control group; Group B, anesthetized rat model group; Group C, anesthetized rat model + PI3K/Akt agonist group; Group D, anesthetized rat model + PI3K/Akt antagonists group; Group E, PI3K/Akt agonist group. The comparison of mRNA expression of PI3K **A.**, AKT **B.**, mTOR **C.** and P70S6K **D.** in cerebral cortex, hippocampus, cerebellum, brain stem, and thalamus of rats among the five groups. The superscript lowercase letters (^a, b, c, d^) represent the pairwise comparison among the five groups with the same protein and tissue. The same superscript lowercase letter indicated the *P* > 0.05, and the different superscript lowercase letter indicated the *P* < 0.05.

As compared to Group A, the mRNA expression of PI3K, AKT, mTOR and P70S6K in the cerebral cortex, brain stem, and thalamus of rats in Groups B, C, D and E were increased (all *P* < 0.05), while no differences were observed in the mRNA expression in hippocampus or cerebellum among the five groups. The mRNA expression of PI3K, AKT, mTOR and P70S6K in cerebral cortex, brain stem, and thalamus of rats in Group C were higher than those in Group B (all *P* < 0.05). In contrast, mRNA expression in these same brain regions was lower in rats from Group D when compared to Group B (all *P* < 0.05). No significant differences were observed between Group E and Group D on the mRNA expression levels of PI3K, AKT, mTOR and P70S6K in cerebral cortex, brain stem and thalamus (Table 5, Figure 6).

**Table 5:** The mRNA expression levels of PI3K-Akt-mTOR pathway proteins in cerebral cortex, hippocampus, cerebellum, brain stem and thalamus of rats among the five groups (`x ± s)

		Cerebral cortex	Hippocampus	Cerebellum	Brain stem	Thalamus
PI3K	Group A	2.07 ± 0.32^a^	2.15 ± 0.23	2.36 ± 0.23	2.41 ± 0.22^a^	2.37 ± 0.21^a^
	Group B	5.14 ± 0.45^b^	2.21 ± 0.21	2.38 ± 0.18	4.67 ± 0.40^b^	5.24 ± 0.45^b^
	Group C	6.37 ± 0.56^c^	2.29 ± 0.18	2.42 ± 0.22	5.53 ± 0.51^c^	6.35 ± 0.58^c^
	Group D	4.22 ± 0.34^d^	2.19 ± 0.16	2.37 ± 0.28	4.29 ± 0.38^d^	4.46 ± 0.36^d^
	Group E	4.08 ± 0.31^d^	2.18 ± 0.17	2.38 ± 0.21	4.32 ± 0.29^d^	4.38 ± 0.41^d^
Akt	Group A	2.25 ± 0.17^a^	2.27 ± 0.20	2.05 ± 0.15	2.26 ± 0.20^a^	2.33 ± 0.19^a^
	Group B	4.39 ± 0.44^b^	2.30 ± 0.21	2.13 ± 0.17	4.50 ± 0.41^b^	4.26 ± 0.42^b^
	Group C	5.78 ± 0.52^c^	2.33 ± 0.17	2.17 ± 0.20	5.72 ± 0.52^c^	5.87 ± 0.47^c^
	Group D	3.47 ± 0.43^d^	2.29 ± 0.18	2.06 ± 0.18	3.38 ± 0.33^d^	3.35 ± 0.33^d^
	Group E	3.53 ± 0.39^d^	2.31 ± 0.19	2.10 ± 0.18	3.43 ± 0.37^d^	3.47 ± 0.38^d^
mTOR	Group A	0.75 ± 0.06^a^	0.78 ± 0.06	0.68 ± 0.06	0.71 ± 0.06^a^	0.65 ± 0.05^a^
	Group B	2.12 ± 0.21^b^	0.81 ± 0.08	0.70 ± 0.05	2.35 ± 0.25^b^	2.17 ± 0.19^b^
	Group C	2.83 ± 0.32^c^	0.83 ± 0.08	0.73 ± 0.07	2.92 ± 0.28^c^	3.01 ± 0.27^c^
	Group D	1.45 ± 0.18^d^	0.79 ± 0.07	0.69 ± 0.05	1.69 ± 0.13^d^	1.38 ± 0.11^d^
	Group E	1.51 ± 0.19^d^	0.80 ± 0.07	0.71 ± 0.06	1.73 ± 0.18^d^	1.46 ± 0.14^d^
P70S6K	Group A	1.47 ± 0.15^a^	1.51 ± 0.11	1.39 ± 0.12	1.45 ± 0.12^a^	1.53 ± 0.10^a^
	Group B	5.18 ± 0.39^b^	1.53 ± 0.14	1.41 ± 0.13	4.93 ± 0.37^b^	5.06 ± 0.46^b^
	Group C	6.21 ± 0.58^c^	1.55 ± 0.12	1.43 ± 0.15	5.82 ± 0.49^c^	6.17 ± 0.57^c^
	Group D	4.14 ± 0.43^d^	1.53 ± 0.10	1.40 ± 0.14	3.78 ± 0.36^d^	3.89 ± 0.40^d^
	Group E	4.19±0.38^d^	1.54 ± 0.11	1.42 ± 0.14	3.83 ± 0.38^d^	4.02 ± 0.43^d^

Note: Group A, blank control group; Group B, anesthetized rat model group; Group C, anesthetized rat model+PI3K/Akt agonists group; Group D, anesthetized rat model+PI3K/Akt antagonists group; Group E: PI3K/Akt agonist group. The superscript lowercase letters (^a^, ^b^, ^c^, ^d^) represent the pairwise comparison among the five groups with the same protein and tissue. The same superscript lowercase letter indicated the *P* > 0.05, and the different superscript lowercase letter indicated the *P* < 0.05.

### Expression of PI3K-AKT-mTOR pathway proteins

**Figure 7:**
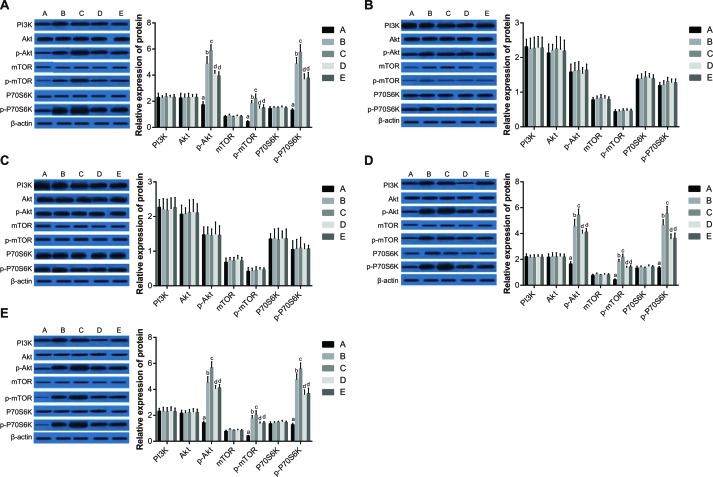
Expression of PI3K-AKT-mTOR pathway proteins in the cerebral cortex **A.**, hippocampus **B.**, cerebellum **C.**, brain stem **D.**, and thalamus **E.** of rats among the five groups. Note: Group A, blank control group; Group B, anesthetized rat model group; Group C, anesthetized rat model + PI3K/Akt agonist group; Group D, anesthetized rat model + PI3K/Akt antagonists group; Group E, PI3K/Akt agonist group.

To further explore the role of the PI3K-AKT-mTOR pathway in the recovery from general anesthesia, the protein expression of PI3K, AKT, p-AKT, mTOR, p-mTOR, P70S6K and p-P70S6K were detected in cerebral cortex, hippocampus, cerebellum, brain stem, and thalamus of rats. There were no differences in the expression of PI3K, AKT, mTOR or P70S6K in cerebral cortex, brain stem, or thalamus among groups. However, as compared to Group A, the expression of p-AKT, p-mTOR and p-P70S6K in cerebral cortex, brain stem, and thalamus of rats in Group B, C, D and E were increased (all *P* < 0.05). No differences were observed in the expression of any of the measured proteins in hippocampus and cerebellum among the five groups. When compared with Group B, the expression of p-AKT, p-mTOR and p-P70S6K in cerebral cortex, brain stem, and thalamus of rats in Group C were increased (all *P* < 0.05). Further, the expression of p-AKT, p-mTOR and p-P70S6K in cerebral cortex, brain stem, and thalamus of rats in Group D were decreased as compared to Group B (all *P* < 0.05). There were no significant differences on the protein expression levels of p-AKT, p-mTOR and p-P70S6K in cerebral cortex, brain stem and thalamus between Group E and Group D (all *P* > 0.05) (Figure 7).

### The results of HE staining

**Figure 8:**
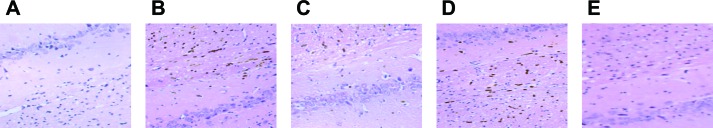
Histopathologic changes in the hippocampal tissue detected by HE staining Note: Group A, blank control group; Group B, anesthetized rat model group; Group C, anesthetized rat model + PI3K/Akt agonist group; Group D, anesthetized rat model + PI3K/Akt antagonists group; Group E, PI3K/Akt agonist group.

In Group A and Group E, the HE staining results showed that the larger number of nerve cells were expressed in the hippocampal CA1 areas, with regular shape, normal structure, plenty cytoplasm, large and round nucleus, integrated nuclear membrane, clear nucleolus and uniformly distributed chromatin. In Group B, some nerve cells were found degenerated and necrotized, with shrinking shape, unclear nucleolus, and enlarged surrounding space with a few inflammatory cells assembled in it. The chromatin was condensed with deep stain. In Group C, a few of nerve cells were found degenerated and necrotized, with shrinking shape. In Group D, a great deal of nerve cells degenerated and necrotized. The surrounding space enlarged, interstitium was swollen, inflammatory cells assembled in it, with irregular shape. Cell membrane reductus and curled, karyopyknosis and karyorrhexis were observed, with crescentic nucleus, and the tigroid body dissolved and chromatin were presented in dark blue or blue-black (Figure 8).

## DISCUSSION

In this study, we established an anaesthetized rat model with ketamine and explored the role of the PI3K-AKT-mTOR pathway on the recovery from general anesthesia. We found that the proportion of δ waves in ECoG in anaesthetized rats treated with PI3K/Akt agonist was decreased, while the proportion of δ waves in ECoG in anaesthetized rats treated with PI3K/Akt antagonists was increased. In the Morris water maze, the EL, as well as the duration and frequency of stay in anaesthetized rats treated with PI3K/Akt agonists were shortened, while those same measures in rats treated with PI3K/Akt antagonists were prolonged. In addition, we also found that the expression of PI3K-AKT-mTOR pathway proteins in normal rats were low, while the expression of p-AKT, p-mTOR and p-P70S6K in cerebral cortex, brain stem, and thalamus of anaesthetized rats treated with PI3K/Akt agonists were increased. These results suggest that the cerebral cortex, brain stem, and thalamus may be the targets of these drugs and that the activation of the PI3K-AKT-mTOR pathway may promote the recovery from general anesthesia and enhance spatial learning and memory.

Additionally, our HE staining results showed several nerve cells after anesthesia were degenerated, necrotized, and enlarged surrounding space with a few inflammatory cells assembled in it, and the chromatin was condensed with deep stain. Further, after transfected with PI3K/Akt antagonists, a great deal of nerve cells degenerated and necrotized, and the surrounding space enlarged, interstitium was swollen, inflammatory cells assembled in it, with irregular shape. HE staining results showed that anesthesia may induce neuronal necrosis in rat hippocampal CA1 areas, and the injection of PI3K/Akt agonists could reduce the cells apoptosis. Ketamine is used as a general anesthetic, which can cause neurodegeneration and/or neuroprotection, and the ketamine is regarded as an important inducer of apoptosis of neural cells [25, 26]. It has been reported that the activation of PI3K and Akt may promote the survival of endothelial cells, inhibit the neurologic damage, reduce the apoptosis of inflammatory cells and suppress the injury of neuron [27, 28]. Further, general anesthesia sometimes activates protein kinase c of brain, which acted as an important regulator of multiple physiological processes like neurotransmitter release, ion channel activity, neurotransmitter receptor desensitization, and the activity of Akt [29, 30]. In this regard, we suspected that neural cells apoptosis induced by ketamine may activate the PI3K-AKT-mTOR signaling pathway, and the neural cells apoptosis may reasonably explain the mechanism for delayed response in spatial learning and memory after ketamine anesthesia.

The PI3K-AKT-mTOR pathway is a classical intracellular signaling pathway, and AKT is the key member that promotes survival after cellular injury [31]. A number of studies have demonstrated that the PI3K-AKT-mTOR pathway and its downstream kinases may promote cell proliferation and inhibit apoptosis, and that AKT activates various protein kinases, which may be closely correlated with ischemia-reperfusion injury [32, 33]. Additionally, the activation of AKT may induce the phosphorylation of proteins that stimulate cell proliferation and suppress apoptosis [13, 15]. It is also clear that this pathway may be involved in the development of various cancers and other diseases [34–37]. General anesthesia may evoke stress response, which will increase the blood glucose concentration [38]. The PI3K-AKT-mTOR pathway may be activated due to high glucose, which may promote the proliferation of neural stem cells [39–41]. Further, PI3K not only affects the levels of intracellular Ca^2+^, but also be indirectly activated by the α_2_ adrenergic receptor [42]. Moreover, Ca^2+^ signaling plays an important role in the cellular response to external stimuli and regulating transmembrane signaling pathways in cells [2, 43]. It is also involved in the biosynthesis and release of neurotransmitters, and extracellular Ca^2+^ flows into nerve cells, which may promote the recovery of physiological function [44]. On the other hand, general anesthetics may alter the transmission of extracellular calcium into neurons, thereby reducing neuronal activity [2, 44].

Moreover, at the beginning of anesthesia, the general anesthesia drugs widely distributed in the neural sites of brain, which may inhibit the brain functions of conscious, nervous motion, learning and memory and so on, and the nerve cells in the brain after anesthesia can quickly recover after anesthetics were eliminated [45, 46]. And the recovery from general anesthesia is usually assessed by monitoring behavioral and physiological signs [47]. The rapid response of anesthesia may cause cognitive dysfunction in experimental animals, and the delayed recovery or incompletely recovery of brain nerve function may result in postoperative cognitive function obstacle [48, 49]. In this study, the proportion of δ waves in ECoG was used to evaluate the depth of anesthesia, the duration of LORR and period of ataxia were assessed the anesthesia time or recovery of motor function, Morris water maze was used to evaluate the spatial learning and memory. In our study, we found that PI3K-AKT-mTOR pathway proteins in normal rats were expressed at low levels, while there was increased expression of p-AKT, p-mTOR and p-P70S6K in brain tissues of anaesthetized rats treated with PI3K/Akt agonists. Moreover, the proportion of δ waves in ECoG was increased, while the EL and the duration and frequency of stay in anaesthetized rats with PI3K/Akt agonists were shortened. These results suggest that the PI3K-AKT-mTOR pathway promotes recovery from general anesthesia and enhances spatial learning and memory. Although we were able to make an association between the PI3K-AKT-mTOR pathway and recovery from general anesthesia, the underlying mechanisms are still incompletely understood; hence, further studies should be conducted to explore the underlying mechanisms.

In conclusion, increased expression of PI3K-AKT-mTOR pathway related proteins may promote the recovery from general anesthesia. Further, activation of the PI3K-AKT-mTOR pathway may enhance spatial learning and memory post-anesthesia.

## MATERIALS AND METHODS

### Animals and grouping

Experiments were performed on 150 specific pathogen-free (SPF) Sprague-Dawley (SD) rats (male, 75; female, 75; 10 weeks old), with a median weight of 207.4 ± 12.8 g, from the Experimental Animal Center of Wuhan University (Wuhan, China; Certificate number: SCXK (E) 2008-0004). The rats were maintained on a 12 h light, 12 h dark cycle with access to food and water *ad libitum*. The room temperature was kept at 23 ± 1°C. Rats were randomly assigned into five groups: (1) blank control group (Group A, *n* = 30); (2) anesthetized model group (Group B, *n* = 30); (3) anesthetized model + PI3K/Akt agonist group (Group C, *n* = 30); (4) anesthetized model + PI3K/Akt antagonists group (Group D, *n* = 30); and (5) PI3K/Akt agonists group (Group E, *n* = 30). All experimental procedures were approved by the Second Hospital of Hebei Medical University and performed in strict compliance with the Guide for Care and Use of Laboratory Animals of the National Institutes of Health [22]. Efforts were exerted to reduce the number of animals used and to minimize their suffering.

### Anesthetized rat model

Electroencephalographic (EEG) electrodes and injection pipes were placed into the lateral ventricles of rats. Experiments were performed in 2 weeks after surgery. Thirty minutes before the experiment, the rats in Group A and Group E were injected intraperitoneally (i.p.) with 2 ml saline, while the rats in Groups B, C and D were injected with 2 ml ketamine (75mg/kg; purchased from Jiangsu Hengrui Medicine Company, Lianyungang, China). Five minutes before the experiment, the rats in Group A and Group B were injected with 10 μl artificial cerebrospinal fluid (ACSF) in the lateral ventricles, while the rats in Group C and Group E were injected with 10 μl IGF-1 (PI3K/Akt agonists, R&D Company, USA) and the rats in Group D were injected with 10 μl LY294002 (PI3K/Akt antagonists, R&D Company, USA). The vital signs of rats, including heart rate (HR), respiratory rate (RR), mean arterial pressure (MAP), arterial partial pressure of oxygen (PaO_2_), partial artery carbon dioxide pressure (PaCO_2_), and arterial oxygen saturation (SaO_2_), were measured in each group.

### Record and analysis of electrocorticogram (ECoG)

After the rats were anesthetized by i.p. ketamine (75 mg/kg), a screw and metal casing were embedded into the frontal lobe and lateral ventricle, respectively and fixed onto the skull with dental cement to record the cortical EEG activity. Afterwards, all rats were given food and water *ad lib.,* and the room temperature was maintained at 23 ± 1°C under a 12h:12h light-dark for two weeks. Data were recorded by a RM6240 biological signal processing system (Cheng Du, Sichuan, China), and then the proportion of δ (0.5~4 Hz) waves in ECoG was analyzed to determine the depth of anesthesia. The ECoG of lateral ventricles 5 min before (for a reference) and after drug administration were measured and analyzed. The change of percent of δ wave before and after drug administration was used to judge the influence of the PI3K-AKT-mTOR pathway on the depth of anesthesia.

### Behavioral measurements

The behaviors of rats were measured through the loss of righting reflex (LORR), which is when the rat cannot recover to the normal standing posture after 5s supine when inverted three times in rapid succession. The duration of LORR is the period between the LORR and the recovery of righting reflex (RORR). Based on the method of Rebuelto [23], the duration of LORR and period of ataxia for anesthetized rats in Group B, C and D were recorded, to judge the inductive effects of i.p. ketamine and the awakening effects of IGF-1 and LY294002.

### Morris water maze

The Morris water maze [24] was used to assess spatial learning and memory. The maze was composed of a pool, recording equipment (camera placed 3 m above the pool) and computer analysis system. The pool was divided into four quadrants, and the platform, 0.5 cm above the water surface, was fixed in the center of the first quadrant. The water temperature was maintained at 25 ± 2°C, with other surrounding environment remaining unchanged. To hide the platform, the platform, tank bottom, and wall of pool were painted black. To acclimate the rats to the maze environment, they were allowed to swim in the pool for 2 minutes without the platform the day before experiment.

The Morris water maze consisted of three steps: (1) Place navigation test (PNT). Rats were put into the pool facing the pool wall from each of the four quadrants. Escape latency (EL), the duration of climbing on the platform was recorded. If a rat didn't find and climb on the platform within 120 s, it was guided to the platform and recorded as 120 s. After rest for 60 s, the rats continued training (4 times/day, for 5 days). (2) Spatial probe test (SPT). The platform was removed after PNT. Rats were put into the pool facing the pool wall from the third quadrant. The frequency of passing the original platform and the duration of stay within that quadrant was recorded for 120 s. The motion curves of all rats were also recorded. (3) Visible platform test (VPT). The platform was placed 2 cm above the water surface, rats placed in the third quadrant as in SPT, and the latency to find the platform was recorded to verify sensory and motor function.

### Sample collection

After the end of Morris water maze, rats were rapidly decapitated and underwent craniotomy on ice. The cerebral cortex, hippocampus, cerebellum, brain stem, and thalamus were collected. The collected samples were divided into two parts: one part of samples was stored in an Rnase-free cryotube, frozen with liquid nitrogen, and saved for RNA extraction and real-time quantitative polymerase chain reaction (RT-qPCR); the other part was transferred into a cryotube, frozen with liquid nitrogen, and saved for total protein extraction and western blotting analysis.

### Detection of mRNA expression of PI3K-AKT-mTOR pathway proteins by RT-qPCR

Total mRNA was extracted using TRIzol Reagent (Invitrogen) following the manufacturer's instructions. The primers for PI3K, AKT, mTOR, P70S6K and β-actin were designed and synthesized by the TaKaPa Company (Dalian, China), and sequences are shown in Table 1. cDNA was generated using a PrimeScript RT Reagent Kit (Takara, Dalian, China) according to the manufacturer's instructions. Real-time quantitative PCR analyses were carried out with SYBR Premix Ex TaqII (Takara, Inc., Dalian, China) using an ABI Prism 7300 system (Life Technologies), and β-actin was used as an internal reference gene. The reverse transcription reaction (50 μl) consisted of 25 μl SYBR Premix Ex TaqII, 2 μl forward primers, 2 μl reverse primers, l μl ROX Reference Dye (50×), 4 μl template cDNA, and 16 μl dH_2_O. PCR reaction conditions were: initial denaturation at 95°C for 10 s and 40 cycles of denaturation at 95°C for 5 s and annealing/extension at 60°C for 31 s. The fluorescence intensity of FAM490 was detected. The mRNA expression of PI3K, AKT, mTOR and P70S6K were detected in cerebral cortex, hippocampus, cerebellum, brain stem, and thalamus.

### Western blotting for the expressions of PI3K-AKT-mTOR pathway proteins

The total protein concentration was determined by bicinchoninic acid (BCA) assay. The protein was separated by sodium dodecyl sulfate-polyacrylamide gel electrophoresis (SDS-PAGE), and then the separated gel was electrophoresed and transferred to nitrocellulose membrane. After blocking with 1% non-fat dry milk at room temperature for 4 h, membranes were incubated with primary antibodies for 3 h. β-actin was used as an internal reference. Following primary antibody incubations, membranes were washed with phosphate buffer solution (PBS), and incubated with secondary antibodies for 2 h at room temperature. The nitrocellulose membrane was washed with PBS at least three times. Gel images were captured using the Gel Documentation System (Bio-Rad, Inc., Hercules, CA, USA) and semi-quantitative analyses of PI3K, AKT, p-AKT, mTOR, p-mTOR, P70S6K, and p-P70S6K in the cerebral cortex, hippocampus, cerebellum, brain stem and thalamus were performed using image-pro PLUS. Goat anti-rat monoclonal antibodies for PI3K, AKT, p-AKT, mTOR, p-mTOR, P70S6K and p-P70S6K were purchased from Santa Cruz Biotechnology (Santa Cruz, USA).

### Histopathologic changes in the hippocampal tissues observed by hematoxylin and eosin (HE) staining

Samples were fixed by formaldehyde and embedded in paraffin. Coronal sections (5 μm) were made with LM1235 paraffin section machine (Leica, German) at the area of suprachiasmatic hippocampus, with dewaxing, embedding and then HE staining. The HE staining process: The paraffin slices were dewaxed to distilled water, as: xylene I for 15 mins, xylene II for 15 mins, anhydrous alcohol I, anhydrous alcohol II, 95% alcohol I, 95% alcohol II, 80% alcohol (dehydration time at all levels were 2~5 mins), and then washed with distilled water; hematoxylin stained for 5 mins and washed; 1% hydrochloric acid alcohol differentiated and washed; returned to blue with ammonia and washed; stained with eosin for 3 mins and washed; dehydrated with ethanol of gradient concentration, as: 80% alcohol, 95% alcohol I, 95% alcohol II, absolute alcohol I, absolute alcohol II (all levels for 2~3 mins); cleared in xylene for 2 times with a total time of 10 mins; wiped out the xylene around the slices, quickly added the neutral gum and sealed the slices with cover glasses. A double-blind method was adopted to observe the morphological changes of the hippocampal tissues under light microscope. Xylene, ethanol, hematoxylin, eosin, and neutral gum were purchased from Sigma, Company, USA. KD-BM paraffin embedding machine were purchased from Jinhua kolno Electronic Technology Co. Ltd., China. CX-31 optical biological microscope was purchased from Olympus Company.

### Statistical methods

SPSS 19.0 statistical software (SPSS Inc., Chicago, IL, USA) was used for statistical analyses. Data are expressed as means and standard deviations (x ± s). One-way analysis of variance (ANOVA) analysis was used for multiple group comparisons. Student's *t* tests were used for comparisons between two groups. *P* < 0.05 was considered statistically significant.
